# Predictors and Outcome of Pericardial Effusion After Hematopoietic Stem Cell Transplantation in Children

**DOI:** 10.1007/s00246-017-1747-x

**Published:** 2017-10-23

**Authors:** A. B. Versluys, H. B. Grotenhuis, M. J. J. Boelens, A. M. C. Mavinkurve-Groothuis, J. M. P. J. Breur

**Affiliations:** 1 Blood and Marrow Transplantation Program, Department of Pediatrics, University Medical Center Utrecht, Utrecht, The Netherlands; 2 Department of Pediatric Cardiology, University Medical Center Utrecht/Wilhelmina Children’s Hospital, PO box 85090, 3508 AB Utrecht, The Netherlands; 3 Princess Máxima Center for Pediatric Oncology, Utrecht, The Netherlands

**Keywords:** Pericardial effusion, Stem cell transplantation, Pericardiocentesis, Echocardiography

## Abstract

Pericardial Effusion (PE) is a potentially life-threatening complication of Hematopoietic Cell Transplantation (HCT). Our study aim was to identify incidence, risk factors, response to treatment, and outcome of PE after pediatric HCT. All patients after HCT at our tertiary center between 2005 and 2010 were included. Endpoints were PE development and overall survival. We analyzed patient factors, HCT details, and complications and used Cox proportional hazard regression modeling to identify predictors for PE. Twelve out of 129 patients (9.3%) developed PE. Multivariate analysis demonstrated that young age at HCT was a predictor for PE: expressed per year increase in age HR = 0.66 (95% CI 0.46–0.95, *p* = 0.03). PE had no impact on overall mortality of HCT. Mild respiratory symptoms and vomiting were presenting symptoms for PE. Discontinuation of calcineurin inhibitors—with or without pericardiocentesis—was the only effective treatment for PE, in contrast to diuretics or increased immunosuppression. Seven of 12 PE patients had pericardiocentesis, which was safe and effective in all. Pericardial effusion is not rare after HCT, and young age is the only significant risk factor. Calcineurin inhibitor toxicity appears to be the primary cause of PE after HCT, and discontinuation is effective in the reduction of PE. Pericardiocentesis for PE is a safe and effective procedure. Pericardial effusion did not have an impact on survival after HCT.

## Introduction

Hematopoietic stem cell transplantation (HCT) is a well-known and effective treatment for both malignant and non-malignant disorders. Unfortunately HCT is associated with high morbidity and mortality rates, mainly due to graft-versus-host disease (GVHD), infections, and toxicity [1–3].

The incidence of early cardiac complications after HCT is estimated between 5 and 10%, including heart failure, acute ventricular fibrillation, and cardiac tamponade occurring in less than 2% of patients [4, 5]. Pericardial effusion (PE) after HCT has a reported incidence between 0.2 and 16.9% [5, 6]. In most case reports, PE is attributed to GVHD, infection, or the toxicity of conditioning or post-transplant immune suppression [4–6]. However, the exact etiology of PE remains unclear in the majority of HCT cases.

Therefore, the objective of this study was to describe the incidence of PE after HCT, to identify predictors for development of PE after HCT, and to evaluate presenting symptoms, diagnosis, and treatment.

## Materials and Methods

### Study Design and Study Population

All patients who underwent allogeneic HCT between January 2005 and 2010 at our tertiary center (*N* = 129) were included and retrospectively studied. Patients were enrolled in the HCT protocol after providing written informed consent for both the HCT and research protocol. This protocol has been approved by the local ethics committee.

### Treatment Protocol

#### Conditioning

Conditioning consisted of a combination of several cytostatic agents or total body irradiation (TBI), based on disease- and patient-specific protocols. Patients aged > 3 years with ALL received TBI (12 Gy in 6 fractions) combined with etoposide. The conditioning in the majority of other cases was Busulfan-based (iv, targeted on AUC of 85–95), combined with either cyclophosphamide (120 mg/kg) or Fludarabine (160 mg/m^2^). Other cytostatic agents, administered less regularly, were Treosulfan, Melphalan, and Thiotepa.

#### Graft-Versus-Host Disease Prophylaxis and Supportive care

Graft-versus-host disease prophylaxis consisted of ciclosporin A (aiming for a trough level of 150–250 µg/L). Methotrexate (10 mg/m^2^ on days 1, 3 and 6) was administered to patients receiving an unrelated bone marrow or peripheral blood stem cell transplant. Methylprednisolone (1 mg/kg/day for 28 days) was given to patients receiving unrelated cord blood transplant. All patients receiving an unrelated donor graft were given antithymocyte globulin serotherapy.

Antimicrobial prophylaxis consisted of daily ciprofloxacin and fluconazole from the start of conditioning until resolution of neutropenia. Cefazolin was administered as additional prophylaxis in the mucositis phase, and cotrimoxazole was given three times a week as Pneumocystis carinii pneumonia prophylaxis, starting 1 month after transplantation. Acyclovir prophylaxis was given in case of positive herpes simplex virus serology. Reactivation of adenovirus, cytomegalovirus, and Epstein–Barr virus was monitored weekly and pre-emptive antiviral therapy was given when indicated. No other antiviral prophylaxis was administered.

### Cardiac Survey

All HCT patients underwent routine cardiac assessment prior to HCT, including electrocardiography and echocardiography, to identify structural cardiac anomalies, the presence of PE, and for evaluation of cardiac function and dimensions. Repeat echocardiography was performed in case of PE prior to HCT, or when clinical signs of cardiac failure, unexplained respiratory distress, or abnormalities in cardiac contour on chest X-ray or CAT-scan after HCT were present. All echocardiography studies were analyzed by an attending pediatric cardiologist.

A quantitative echocardiographic assessment reported by D’Cruz et al. was used to estimate the volume of PE by two-dimensional echocardiography [7]. Expiratory early-diastolic right ventricular (RV) free wall collapse and expiratory late-diastolic right atrial (RA) collapse were recorded to assess for cardiac tamponade [7]. In addition, atrial collapse was quantified by calculation of a bi-atrial transverse–cardiac internal transverse ratio [7]. Bi-atrial and cardiac internal transverse dimensions were measured and a cut-off ratio indicative of cardiac tamponade of < 0.85 was used [7]. The presence of a swinging heart (beat-to-beat swinging of the heart in the pericardial sac due to cardiac tamponade) was qualitatively scored. Mitral (MV) and tricuspid (TV) valve inflow variations during inspiration were recorded to assess for cardiac tamponade. A MV E-wave decrease by > 30% and a TV E-wave decrease by > 50% during inspiration when compared during expiration were considered indicative of cardiac tamponade [7].

### Definitions of Disease

Pericardial effusion was defined as PE on echocardiography, with a recognizable accumulation of fluid between the visceral and parietal pericardium. When baseline cardiac survey already revealed PE, patients were only regarded to have developed PE in case of an increase in PE after HCT.

A matched donor was defined as a molecularly typed 10 out of 10 human leukocyte antigen (HLA) match for bone marrow/peripheral blood stem cell grafts, and 6 out of 6 matched cord blood grafts based on intermediate resolution testing for HLA-A and HLA-B, and high-resolution testing for HLA-DR [8].

Veno-occlusive disease (VOD) was defined according to the Baltimore criteria: weight gain > 5%, tender hepatomegaly, ascites, and increased bilirubin > 34.2 µmol/L. VOD was treated with defibrotide 25 mg/kg/day for at least 2 weeks.

Uremia was defined as serum urea concentration of > 10.0 mmol/L, lasting for more than 7 days.

### Treatment Algorithm of Pericardial Effusion

All HCT patients with PE were treated with diuretics. Depending on differential diagnosis (alloimmunity, infection or toxicity), immunosuppressive agents were increased, antibiotics were switched, or drugs with PE as potential side effect were stopped or switched. Increase in immunosuppressive therapy consisted of prednisolone 2 mg/kg/day for 1–2 weeks or a methylprednisolone-pulse 10 mg/kg/day for 3 days. After this period, prednisolone was gradually tapered, depending on clinical symptoms. Pericardiocentesis or surgical drainage were performed when indicated, either clinically or by echocardiography.

### Endpoints

The primary endpoint of this study was the development of echocardiographically proven PE after HCT. The secondary endpoint was overall survival of HCT. To analyze risk factors for the primary and secondary endpoints, we considered variables associated with the recipient (age at HCT, gender), underlying disease (malignant vs. non-malignant), the donor and transplantation techniques (cell source, donor relationship, HLA disparity, conditioning regimen), and HCT complications (acute/chronic GVHD, VOD, and uremia).

### Statistical Analysis

All statistical analyses were performed using SPSS 21 (SPSS Inc, Chicago, United States). Differences between the PE group and the non-PE group were tested using Pearson’s *χ* ^2^-test. Linear regression was done for time-independent variables and Cox proportional hazard models. Results are expressed as Hazard ratios (HRs) or Odds ratio (OR) and corresponding 95% confidence intervals. Results with a *p* value < 0.05 and confidence intervals not including 1.00 were considered statistically significant.

## Results

### Patient Characteristics

A total of 129 patients after allogeneic HCT were studied with median age at HCT of 5.2 years (range 0.16–21.2 years). Baseline characteristics are shown in Table 1.

**Table 1:** Patient characteristics

Characteristics	*N*
Patients	129
Gender	
Male	71
Female	58
Age at HSCT [in years, median (range)]	5.2 (0–21)
Disease	
Malignant indications	71
Acute lymphoblastic leukemia	30
Acute myelogenous leukemia	19
Other	22
Non-malignant indications	58
Immunodeficiencies	27
Inborn errors of metabolism	31
HLA disparity	
Related	33
MUD*	59
MMUD**	37
Graft source	
Cord blood	55
PBSC/BMT	74
Conditioning	
TBI-based	26
Busulfan-based	103
PE pre-SCT	20

A matched donor was defined as a molecularly typed 10 out of 10 match for BM/PBSC grafts and 6 out of 6 matched CB grafts based on intermediate resolution (HLA-A and HLA-B on serology and HLA-DR on high resolution)

* Matched unrelated donor

** Mismatched unrelated donor

### Primary Endpoint: Pericardial Effusion

Twelve patients (9.3%) developed PE after HCT. The median time to development of PE after HCT was 79 days (range 28–230 days). The median age of patients developing PE was 1.1 years (0.3–7.6 years). All HCT patients with PE presented with vomiting and mild respiratory symptoms. Despite these mild symptoms, echocardiographic evidence of cardiac tamponade was present in 7 of 12 PE patients.

Table 2 shows patient characteristics, echocardiographic findings, therapies administered, and outcome. Predictors for PE are shown in Table 3. Univariate analysis only showed a significant association between PE and age (HR 0.18, 95% CI 0.05–0.63, *p* value < 0.01). Multivariate analysis confirmed age at HCT as a statistically significant factor with decrease in age per year: HR 0.66 (95% CI 0.46–0.95, *p* value 0.03). Estimated pericardial effusion by echocardiography did not correlate with drained volume by pericardiocentesis (Table 2, *p* = 0.48), although the low number of available drained-volume measurements may have obscured our findings. Estimated nor drained pericardial effusion volumes did correlate with any of the echocardiography parameters associated with cardiac tamponade, i.e., presence of expiratory early-diastolic RV free wall collapse, expiratory late-diastolic RA collapse, a swinging heart, the bi-atrial transverse–cardiac internal transverse ratio, the bi-atrial and cardiac internal transverse dimensions (tamponade if < 0.85), and MV and TV inflow variation (*p* > 0.05 for all).

**Table 2:** Characteristics of patients who developed PE after HSCT

Patient	♂/♀	Disease	Age at transplant (years)	Days to PE	Stop of calcineurin inhibitors (yes/no, days after PE)	Significant flow patterns	Estimated effusion		Pericardiocentesis (yes/no, immediate/delayed)	Drained volume (mL)	Outcome (alive/death; cause)
							Ml	mL/kg			
1	♀	SCID	4 months	43	Yes, 23	RV collapse, RA collapse, TV: E-wave increase > 50% during inspiration	62	14, 1	Yes Delayed	200	Alive
2	♂	MPS type I	1	86	No	RV collapse, RA collapse, TV: E-wave increase > 50% during inspiration, ratio < 0.85	108	10, 5	No	NA	Alive
3	♀	Osteopetrosis	3, 5 months	47	Yes, 1	None	13	3, 1	No	NA	Death: due to underlying disease
4	♀	JMML	1	112	Yes, 6	RA collapse, MV > 30% variation, TV > 50% during inspiration, ratio < 0.85	55	6, 5	Yes Delayed	NR	Alive
5	♀	MPS type I	1	230	Yes, 1	RV collapse, TV > 50% during inspiration, swinging heart	112	10, 6	Yes Delayed	95	Alive
6	♀	SCID	9 months	64	No	RV collapse, RA collapse, ratio < 0.85, Swinging heart	103	21, 0	Yes Immediate	150	Alive
7	♂	AML	3	93	No	None	111	6, 5	Yes Immediate	110	Death; unexplained sudden death
8	♀	HLHC	6	31	Yes, 69	RA collapse, TV > 50% during inspiration, ratio <0.85	393	15, 1	No	NA	Death; infection-related
9	♂	ALL	7, 5	28	No	None	72	3, 1	No	NA	Alive
10	♀	ALL	2, 5	56	Yes, 13	RV collapse, RA collapse, ratio < 0.85, Swinging heart	227	15, 7	Yes Delayed	225	Alive
11	♂	β-thalassaemia	10 months	68	Yes, 5	RV collapse, MV > 30% variation, TV > 50% during inspiration, swinging heart	262	28, 8	Yes Immediate	280	Alive
12	♀	SCID	6 months	87	Yes, 5	MV > 30% variation, TV: E-wave increase > 50% during inspiration	77	10, 7	No	NA	Alive

*NA* not applicable, *NR* not recorded, *SCID* severe combined immunodeficiency, *MPS* mucopolysaccharidosis, *JMML* juvenile myelomonocytic leukemia, *HLHC* hemophagocytic lymphohistiocytosis, *AML* acute myeloid leukemia; *ALL* acute lymphoblastic leukemia

**Table 3:** Statistical analysis of predictors for pericardial effusion after HSCT

	Total, *N*	HR	95% CI	*p* value
Univariate analysis				
Non-malignant indication	58	2.88	0.82–10.09	0.1
Cell source				
CB*	59	2.79	0.57–13.77	0.21
MUD**	37	0.43	0.04–4.98	0.5
Male gender	71	0.77	0.24–2.54	0.67
Age (increase per year)		0.66	0.47–0.93	0.02
Busulfan-based conditioning	103	0.96	0.19–4.80	0.96

	Total, *N*	OR	95% CI	*p* value
aGVHD	48	0.42	0.09–1.99	0.27
VOD	27	0.47	0.57–3.85	0.48

	Total, *N*	HR	95% CI	*p* value
Multivariate analysis				
Age (increase per year)		0.66	0.46–0.95	0.03
Non-malignant indication	58	0.91	0.21–3.98	0.89

* Cord blood

** Matched unrelated donor

### Secondary Endpoint: Overall Survival

Overall survival in this HCT cohort was 63%. Four of 12 PE patients (33%) died because of pulmonary fibrosis after pulmonary bleeding, unexplained acute cardiac cause, and EBV post-transplant lymphoproliferative disease (EBV-PTLD), respectively. PE had no influence on mortality (HR 1.45; *p* value 0.23).

### Treatment

**Fig. 1:**
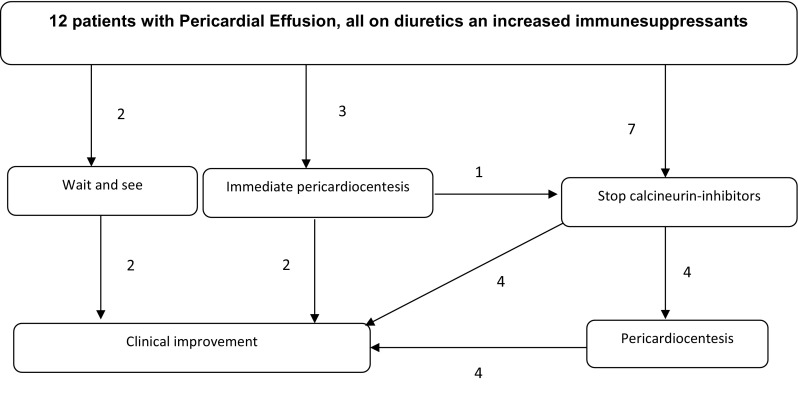
Overview patients with pericardial effusion after HSCT and their treatment

Figure 1 shows treatment and follow-up of the 12 children with PE. Immune suppression was increased in all 12 patients, because of our hypothesis at that time that PE was a symptom of alloimmunity. All PE patients received diuretics. In three PE patients, pericardiocentesis was performed immediately given the severity of PE presentation on echocardiography. In the nine other PE patients, diuretics and increase of immunesuppression was ineffective, and only discontinuation of calcineurin inhibitors resulted in the amelioration of PE. Pericardiocentesis proved necessary in four other patients due to insufficient response to pharmacological treatment. Pericardiocentesis was effective and safe in all patients, without reoccurrence of PE after drainage. Ultimately, only five PE patients were solely treated pharmacologically. Pericardiocentesis demonstrated a transudate with negative microbial cultures in all, and no specific abnormalities on histology.

## Discussion

Pericardial effusion is not an infrequent complication after HCT, with an incidence of 9.3% in our HCT series. Younger age at time of HCT appears to be the only predictor for the development of PE. Although PE is a severe and potentially life-threatening complication, all PE patients could sufficiently be treated by discontinuation of calcineurin inhibitors with or without pericardiocentesis. The occurrence of PE after HCT did not influence survival.

In a large adult HCT series only 0.2% of 2821 patients developed PE or cardiac tamponade [5]. The incidence of PE after HCT appears to be higher in children (incidence between 4.4 and 19%), with a wide variety of outcome and associations [7–10]. Rhodes et al. studied 205 children undergoing HSCT and clinically significant PE was identified in nine patients (4.4%) without PE-associated deaths [11]. Pericardial effusion developed at a median of 30 days after HSCT and all patients had acute GVHD at time of PE diagnosis, suggesting an association with alloimmunity and a place for increase in immunosuppressive therapy [11]. The majority of patients (7 of 9; 78%) required pericardiocentesis. Neier et al. found PE in 16.9% of 158 pediatric HSCT recipients and multivariate analysis identified older age at HSCT, high-risk disease prior to HSCT, allogeneic transplantation, myeloablative conditioning, and TBI as significant factors for the development of PE [6]. Interestingly, pericardial effusion was found to be an important risk factor for mortality [6]. Aldoss et al. [9] describe an incidence of PE of 19% in 296 children receiving allogeneic HCT. Risk factors for the development of PE were myeloablative conditioning, CMV positivity of recipient, and prolonged neutropenia. This might support an infectious etiology, although no infectious agents were recovered from the effusion [9].

The etiology of PE after HSCT remains unclear. In most reports PE is attributed to either toxicity of conditioning or GVHD [5–11]. A recent report suggests an association between transplant-associated thrombotic microangiopathy (TA-TMA) and PE [10]. TA-TMA is a known side effect of calcineurin inhibitors, and is also treated by withdrawing calcineurin inhibitors and sometimes by giving diuretic therapy [10]. Pericardial effusion is less likely caused by direct toxicity of conditioning regimen since the median time to develop PE after HSCT was 78 days and adverse events of conditioning tend to occur earlier after HSCT. In our study, young age was found to be the only predictor for PE after HSCT, which has not been reported for the pediatric HSCT population before. In contrast, Neier et al. report older age as a risk factor [6], which might be explained by the fact that they have analyzed a different cohort of HSCT patients, with relatively more malignant disease, a significant proportion of autologous transplant recipients, a more reduced intensity conditioning regimen and tacrolimus-based aGVHD prophylaxis instead of ciclosporin A-based prophylaxis. Adult papers have also reported an increased age as risk factor for PE after HSCT [7, 12–14], although different underlying diseases, smaller age-differences, and a different impact of increasing age in the elderly pose difficulty to translate adult results to the pediatric HSCT population.

Discontinuation of calcineurin inhibitors appeared to be the only effective conservative measure to improve PE. Increasing immune suppression was ineffective in our cohort, and no association was found between GVHD and PE. Therefore, calcineurin inhibitor toxicity appears to be the primary cause of PE after HSCT, which is in line with more recent reports [10]. Calcineurin inhibitors have a proven effect on the secretion of endothelin I and other pericardial/myocardial derived factors, and changes in these factors might increase vascular permeability of the serous pericardium [15]. Another hypothesis could be the proinflammatory effect of calcineurin-associated TA-TMA leading to serositis and fluid accumulation in the pericardial space [15].

Pericardiocentesis appears to be a safe and effective treatment of PE leading to cardiac tamponade. This finding is supported by a recent case series report [15]. Overall mortality was 37% in our study. In the PE group 33% of children died, while no deaths were attributable to PE (e.g., cardiac tamponade). This firmly contrasts with the mortality rate of 82.6% in pediatric cases with PE as published by Neier et al. [6]. In their study, PE was the most significant risk factor for death after HSCT, although none of their PE patients died directly of pericardial tamponade or surgical intervention [6]. Their treatment regimen of PE was not described, so it is unclear whether or not additional treatment for PE could have influenced overall outcome resulting in PE.

All PE patients in our study presented with mild respiratory symptoms and vomiting, indicating that PE should be considered in patients within the first months after HSCT with relatively mild symptoms like tachypnea and dyspnea. The severity of clinical symptoms did not determine outcome or the severity of PE.

**Fig. 2:**
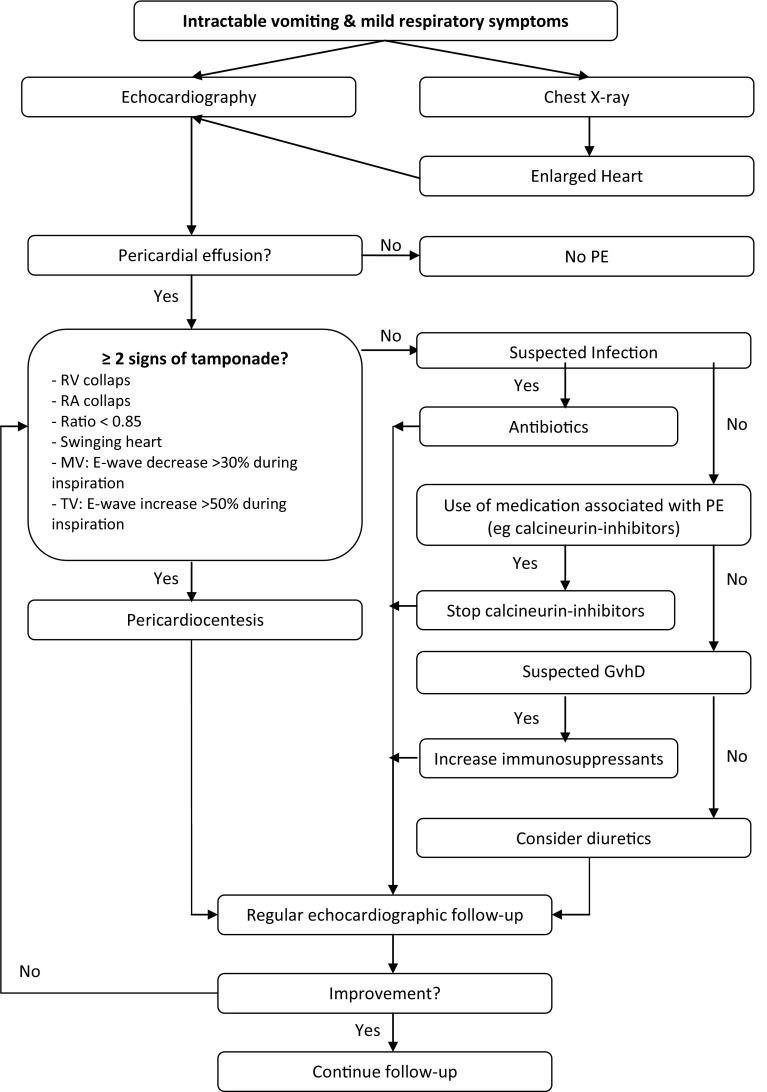
Flow chart: diagnostics and treatment protocol PE after HSCT

None of the used echocardiographic parameters were predictive of the need of pericardiocentesis or outcome, although this may have been caused by the small group of PE patients. Two-dimensional echocardiography has been used to estimate the total amount of PE in pediatric patients by a method first described in adults by D’Cruz et al. [7]. The estimated amount of PE has been reported to correlate relatively well to the amount of PE obtained at pericardiocentesis in older children (> 1 year of age) [7]. However, in our series the estimated volume of PE by echocardiography did not correlate with the volume of PE drained by pericardiocentesis. In addition, although the amount of PE may be predictive for cardiac tamponade, in our study we did not find any relation between amount of PE (corrected for body weight) and cardiac tamponade. Therefore, echocardiographic evaluation should repeatedly be performed during follow-up, to evaluate the effect of pharmacological treatment and to determine the optimal timing of pericardiocentesis. We hypothesize that the time it takes for PE to develop must be important, since the pericardium is able to adapt to a slow increase in PE over a limited amount of time. Our experience with diagnosis and treatment of PE after HSCT has resulted in a treatment protocol, to be tested prospectively (Fig. 2). The protocol is based on our finding that calcineurin toxicity is the most important cause for PE after HSCT.

## Limitations

Our HSCT cohort of 129 patients is one of the largest pediatric series analyzed to determine risk factors and prognostic factors for PE after HSCT. However, our group is relatively small for (multivariable) statistical analysis. This study was performed retrospectively, which may have caused both bias and confounding. Another limitation of this study is the absence of standardized post-HSCT echocardiographs. Given this limitation, we may have underestimated the number of patients with asymptomatic PE, as well as the timing of the onset of PE.

## Conclusion

In conclusion, pericardial effusion frequently occurs after HSCT and should be suspected in every HSCT patient presenting with unexplained respiratory distress or vomiting. Calcineurin inhibitor toxicity appears to be the primary cause for PE after HSCT and younger age appears to be highest risk factor. Pericardiocentesis provides a safe and effective therapeutic option in patients unresponsive to (changes in) medication, or with echocardiographic/clinical signs of cardiac tamponade. Pericardial effusion did not have an impact on survival after HSCT.
